# Risk Factors for *Elizabethkingia* Acquisition and Clinical Characteristics of Patients, South Korea

**DOI:** 10.3201/eid2501.171985

**Published:** 2019-01

**Authors:** Min Hyuk Choi, Myungsook Kim, Su Jin Jeong, Jun Yong Choi, In-Yong Lee, Tai-Soon Yong, Dongeun Yong, Seok Hoon Jeong, Kyungwon Lee

**Affiliations:** National Health Insurance Service Ilsan Hospital, Goyang, South Korea (M.H. Choi);; Yonsei University College of Medicine, Seoul, South Korea (M.H. Choi, M. Kim, S.J. Jeong, J.Y. Choi, I.-Y. Lee, T.-S. Yong, D. Yong, S.H. Jeong, K. Lee)

**Keywords:** *Elizabethkingia anophelis*, *Elizabethkingia miricola*, *Elizabethkingia meningoseptica*, mechanical ventilator, contamination source, South Korea, bacteria, antimicrobial resistance, acquisition

## Abstract

*Elizabethkingia* infections are difficult to treat because of intrinsic antimicrobial resistance, and their incidence has recently increased. We conducted a propensity score–matched case–control study during January 2016–June 2017 in South Korea and retrospectively studied data from patients who were culture positive for *Elizabethkingia* species during January 2009–June 2017. Furthermore, we conducted epidemiologic studies of the hospital environment and mosquitoes. The incidence of *Elizabethkingia* increased significantly, by 432.1%, for 2016–2017 over incidence for 2009–2015. Mechanical ventilation was associated with the acquisition of *Elizabethkingia* species. Because *Elizabethkingia* infection has a high case-fatality rate and is difficult to eliminate, intensive prevention of contamination is needed.

The genus *Elizabethkingia* comprises glucose-nonfermenting, gram-negative rods that are widely distributed in natural environments, including in soil and freshwater, and in hospital environments (*1*). *E. meningoseptica* (originally named *Chryseobacterium meningoseptica*) has been associated with opportunistic infections, such as sepsis in immunocompromised patients and meningitis in neonates (*2*). Two new species of *Elizabethkingia* have been proposed: *E. miricola*, which was first isolated from water from the Russian space station MIR in 2003 (*3**,**4*); and *E. anophelis,* which was first isolated from the midgut of the *Anopheles gambiae* mosquito in 2011 (*5*). Because *E. anophelis* was the most frequently isolated *Elizabethkingia* species in recent clinical studies, as confirmed by 16s rRNA gene sequencing (*6**,**7*), but is commonly misidentified as *E. meningoseptica*, many previously reported cases of *E. meningoseptica* could actually have been caused by *E. anophelis* (*8**,**9*).

Infection caused by *Elizabethkingia* species is difficult to treat and results in a high case-fatality rate, probably because of intrinsic antimicrobial resistance (*10*). *E. meningoseptica* has been documented to carry class A extended-spectrum β-lactamases and 2 chromosomal metallo-β-lactamases (*11**,**12*).

Some outbreaks of *Elizabethkingia* species have been reported to have resulted from a contaminated water source (*13**–**15*). Furthermore, recent increases in the annual incidence of *Elizabethkingia* species. have been reported in many countries (*14**,**16**–**19*). However, knowledge about host risk factors associated with the acquisition of *Elizabethkingia* species is lacking, and no evidence exists that mosquitoes or other sources act as vectors in transmitting it to humans. Thus, we investigated the annual incidence and clinical characteristics of *Elizabethkingia* acquisition in a tertiary teaching hospital in Seoul, South Korea. We aimed to determine whether the incidence of *Elizabethkingia* species had increased in this hospital and to analyze the risk factors associated with *Elizabethkingia* acquisition. To identify the source of *Elizabethkingia*, we obtained and analyzed epidemiologic studies from the hospital environment and mosquitoes.

## Methods

### Study Participants

We retrospectively collected data from all nonduplicate persons who had positive culture results for *Elizabethkingia* species from Severance Hospital, a >2,000-bed tertiary teaching hospital in South Korea, during January 1, 2009–June 30, 2017. The hospital had 10 intensive care units (ICUs) for adults and 2 for children. During this period, the annual number of inpatient-days was >670,000.

Because we had identified strains in our previous study (*7*), we updated our in-house library of matrix-assisted laser desorption/ionization time-of-flight mass spectrometry (MS) (Bruker Daltonic GmbH, https://www.bruker.com). *Elizabethkingia* species were identified by 2 matrix-assisted laser desorption/ionization time-of-flight MS systems; the Bruker MS used the updated in-house library and the Vitek MS (bioMèrieux, https://www.biomerieux.com) used the latest version of IVD (in vitro diagnostic database) V3.2. Strains with discrepant results were confirmed by 16S rRNA gene sequencing using universal primers.

We collected the following clinical data using electronic medical records: age-adjusted Charlson comorbidity index (*20*), sex, sites of specimen collection, date of specimen collection, date of patient death, pulse rate, oxygen saturation, body temperature, chest radiograph results, and any antimicrobial agents administered during hospitalization. We also obtained available laboratory findings from the same day as specimen collection and within 7 days from the same day as specimen collection, including C-reactive protein level, erythrocyte sedimentation rate, and leukocyte count. The Institutional Review Board at Severance Hospital, affiliated with Yonsei University Health System (2017–2101–001), approved this study.

### Epidemiologic Study of Environmental Sources and Mosquitoes

**Figure 1:**
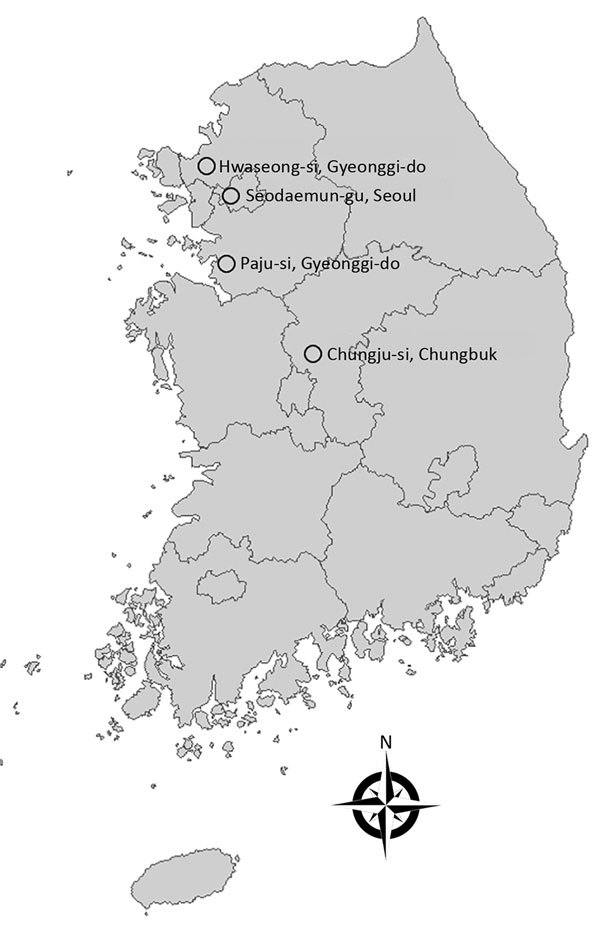
Rural areas of South Korea (Hwaseong-si, Gyeonggi-do; Paju-si, Gyeonggi-do; and Chungju-si, Chungbuk) where adult mosquitoes were collected during July–September 2017 and the urban location of the tertiary teaching hospital (Seodaemun-gu, Seoul) where the study of *Elizabethkingia* infection in patients was conducted during January 2009–June 2017.

We obtained extensive surveillance cultures of 281 common environmental sources by swab culture of equipment and surfaces within patient rooms, restrooms, nursing stations, electronics, furniture, patient care devices, patient transport carts, sinks, and water taps. Additionally, during July–September 2017, we collected adult mosquitoes in 1 urban site (Seodaemun-gu, Seoul, where Severance Hospital is located) and 3 rural areas (Hwaseong-si, Gyeonggi-do; Paju-si, Gyeonggi-do; and Chungju-si, Chungbuk, where annual zoonotic disease monitoring had taken place for their dense population of animal farms) (Figure 1). Mosquitoes were collected using Insect Light Traps Model SR-2000 (Sin Young Inc., Seoul, South Korea) and identified under a stereomicroscope after cold anesthesia, as in the previous study (*21*).

All swab samples and midguts of mosquitoes were inoculated on sheep blood agar and MacConkey agar and incubated overnight. DNA was extracted from mosquitoes using a simple boiling method, and PCR was performed using *Elizabethkingia* species–specific primers (forward, GAACACGTGTGCAACCTGCC; reverse, TCCAGCCACTTCAACCTTAC) and the following cycle parameters: 95°C for 5 min, followed by 35 cycles of 95°C for 30 s, 58°C for 2 min, and 72°C for 1 min; followed by a final extension step at 72°C for 7 min (*22*).

### Pulsed-Field Gel Electrophoresis

We conducted pulsed-field gel electrophoresis (PFGE) analysis of XbaI-digested isolated chromosomal DNA from a total of 12 strains isolated from the environment (7 *E. anophelis*, 3 *E. miricola*, 2 *E. meningoseptica*) and 54 stored strains isolated from inpatients since 2017. PFGE patterns were analyzed using the CHEF DR II system (Bio-Rad, http://www.bio-rad.com) as previously described (*9*).

### Definition

We defined true pathogen cases according to the definitions of Moore et al. and the Centers for Disease Control and Prevention. In brief, we defined these cases as patients with the monomicrobial acquisition of *Elizabethkingia* species and 1 of the following parameters within 2 days before and after acquisition without any other recognized cause: body temperature <36°C or >38°C, pulse rate >90 beats/min (reference range 60–100 beats/min), leukocyte count <4 or >12 × 10^9^ cells/L (reference range 4.0–10.9 × 10^9^ cells/L), C-reactive protein >100 mg/L (reference range 0–8 mg/L), or chest radiography showing new pulmonary infiltrations (*13**,**23*). According to previous studies, outbreaks are determined by whether they exceed 2 SD of the previous disease incidence (*24*,*25*).

### Propensity Score–Matched Analysis

After 2016, the incidence of isolated *Elizabethkingia* species increased significantly. To analyze the increased incidence, we conducted surveillance culture study and compared clinical characteristics of patients who acquired *Elizabethkingia* species before and after 2016. In both the surveillance culture study and the statistical analysis, only 3 study wards showed positive results for the acquisition of *Elizabethkingia*: an ICU (ICU 1, 18 beds) used for cardiovascular disease, an isolation ward (ward A, 50 beds) used for patients with vancomycin-resistant *Enterococcus*, and a general ward (ward B, 50 beds) used for pulmonary disease patients. A total of 6,583 patients have been hospitalized in these wards since 2016.

To adjust confounding factors for the acquisition of *Elizabethkingia* species, we conducted a propensity score (PS)–matched case–control study. We defined case-patients as patients with *Elizabethkingia* species isolated from any clinical specimens during January 2016–June 2017 in ICU 1, ward A, or ward B and control patients as patients without *Elizabethkingia* species in these 3 study wards. We selected 3 variables—hospital ward (p<0.001), period of admission (p = 0.041), and length of stay in the 3 study wards (p<0.001)—for adjustment by univariate analysis (*26**,**27*). We estimated a PS for the predicted probability of acquisition of *Elizabethkingia* species in each patient using the logistic regression model. Then we performed a PS-matched analysis by attempting to match case-patients and control patients (1:3 match) using the nearest-neighbor-matching method. A match occurred when the difference in the logits of the PS was <0.2 times the SD of the scores.

### Statistical Analysis

We assessed all variables using the Shapiro-Wilk test to evaluate Gaussian distributions. Descriptive statistics are presented as a median and interquartile range (IQR) for continuous variables or numbers and percentage for categorical variables. Comparisons between groups were analyzed using the Mann-Whitney U test for continuous variables and the Fisher exact test for categorical variables.

Using conditional logistic regression, we conducted univariate and multivariate regressions between case-patients and control patients of the 3 study wards. Dependent variables included in the multivariate analysis were selected based on statistical significance provided by univariate analysis. Incidence rate ratios and 95% CIs were calculated by comparing the mean incidences between 2009–2015 and 2016–2017 by Poisson regression.

All reported p values are 2-tailed, and p values <0.05 indicate statistical significance. We conducted statistical analyses using R statistical software (R Studio, Inc., https://www.r-project.org).

## Results

**Figure 2:**
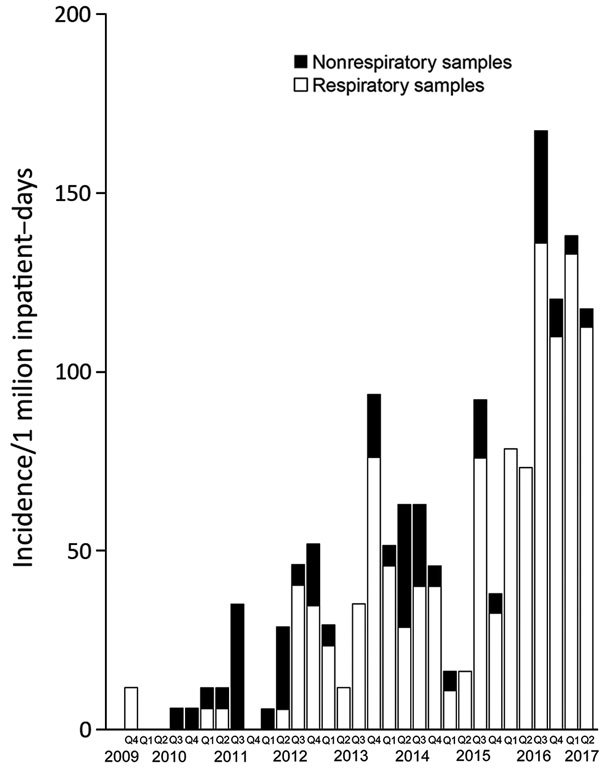
Trends in the quarterly incidence of *Elizabethkingia* infection or colonization in a tertiary teaching hospital, Seoul, South Korea, January 2009–June 2017. Q1, January–March; Q2, April–June; Q3, July–September; Q4, October–December.

The annual incidence of *Elizabethkingia* acquisition in Severance Hospital increased in 2011 (Table 1; Figure 2). According to the defined threshold, years with incidence >2 SD were 2011, 2012, 2013, 2014, and 2016. In particular, incidence increased most significantly to 109.82 cases/1 million inpatient-days in 2016 (p<0.001). An additional 50 cases were reported during January–June 2017, which corresponded to 127.79 cases/1 million inpatient-days. The acquisition incidence of *Elizabethkingia* species increased significantly, by 432.1%, during 2016–2017 over the acquisition incidence during 2009–2015 (relative risk [RR] 4.17, 95% CI 3.28–5.29; p<0.001), mainly because of the increase in strains isolated from respiratory specimens (incidence rate ratio 3.22, 95% CI 2.46–4.20; p<0.001).

**Table 1:** Annual incidence and characteristics of *Elizabethkingia* acquisitions at a tertiary teaching hospital, Seoul, South Korea

Characteristic	2009	2010	2011	2012	2013	2014	2015	2016	2017 Jan–Jun
No. cases	2	2	10	23	29	39	30	84	50
Incidence									
Per 1 million inpatient-days	2.93	2.98	14.60	33.14	42.43	55.74	40.66	109.82	127.79
Per 1,000 inpatients	0.02	0.02	0.10	0.23	0.30	0.39	0.28	0.75	0.88
Sample type, no., may be multiple									
Respiratory	2	0	2	14	25	27	26	76	48
Blood culture	0	2	2	2	2	3	1	4	3
Urine culture	0	0	5	2	2	3	1	1	0
Other*	0	0	1	5	1	7	2	6	1
Species, no.									
* E. anophelis*	1	2	2	7	16	17	15	45	34
* E miricola*	0	0	5	1	4	5	11	25	12
* E. meningoseptica*	1	0	3	3	5	4	1	4	2
Unconfirmed†	0	0	0	12	4	13	3	10	2

*Includes 12 from body fluids, 4 wound swabs, 3 catheter tips, 2 oral swabs, 1 eye swab, and 1 ear swab. †Includes strains that were not stored for identification.

We identified 269 patients who acquired *Elizabethkingia* species during January 2009–June 2017, of whom 134 (49.8%) were reported during 2016–June 2017 (Table 2). Patients who acquired *Elizabethkingia* during 2016–June 2017 were more frequently classified as having contracted a nosocomial infection than were patients who acquired *Elizabethkingia* during 2009–2015. The number of cases in the 3 study wards increased significantly but not in the other wards. In addition, more patients with chronic pulmonary disease or diabetes mellitus were seen during 2016–2017. More patients had a history of mechanical ventilation, a longer length of hospital stay, and a history of steroid use during 2009–2015 than during 2016–2017.

**Table 2:** Baseline characteristics of patients in a tertiary teaching hospital who acquired *Elizabethkingia* and differences before and after 2016, Seoul, South Korea*

Characteristic	Total, n = 269	2009–2015, n = 135	2016–June 2017, n = 134		p value
Median age, y (range)	64.0 (47.0–74.0)	65.0 (46.0–73.0)	63.0 (47.0–74.0)		0.67
Sex, no. (%)					0.24
M	165 (61.3)	88 (65.2)	77 (57.5)		
F	104 (38.7)	47 (34.8)	57 (42.5)		
Nosocomial infection, no. (%)	254 (94.4)	122 (90.4)	132 (98.5)		0.01
Median Charlson comorbidity index (range)	5.0 (3.0–7.0)	5.0 (3.0–7.0)	5.0 (4.0–7.0)		0.04
Comorbidities, no. (%)†					
Solid-organ tumor	86 (32.0)	46 (34.1)	40 (29.9)		0.54
Diabetes mellitus	74 (27.5)	28 (20.7)	46 (34.3)		0.02
Chronic pulmonary disease	70 (26.0)	18 (13.3)	52 (38.8)		<0.01
Chronic kidney disease	54 (20.1)	22 (16.3)	32 (23.9)		0.16
Hemiplegia	37 (13.8)	14 (10.4)	23 (17.2)		0.15
Hematologic malignancy	33 (12.3)	15 (11.1)	18 (13.4)		0.69
Cerebrovascular disease	31 (11.5)	12 (8.9)	19 (14.2)		0.24
Dementia	28 (10.4)	12 (8.9)	16 (11.9)		0.54
Connective tissue disease	23 (8.6)	13 (9.6)	10 (7.5)		0.68
Mild liver disease	13 (4.8)	6 (4.4)	7 (5.2)		0.99
Clinical conditions					
Median hospitalization day of acquisition (range)	33.0 (17.0–69 .0)	28.0 (14.0–55.5)	36.0 (18.0–75.0)		0.02
Median length of hospitalization, d (range)	69.0 (39.0–133.0)	59.0 (35.0–99.5)	78.0 (41.0–149.0)		0.04
Mechanical ventilation, no. (%)	214 (79.6)	99 (73.3)	115 (85.8)		0.02
Steroid use, no. (%)	161 (59.9)	72 (53.3)	89 (66.4)		0.04
Prior ICU stay, no. (%)	142 (52.8)	73 (54.1)	69 (51.5)		0.76
Sample type, no. (%)†					
Respiratory	219 (81.4)	95 (70.4)	124 (92.5)		<0.01
Blood culture	19 (7.1)	12 (8.9)	7 (5.2)		0.35
Urine culture	14 (5.2)	13 (9.6)	1 (0.7)		<0.01
Other	23 (8.6)	16 (11.9)	7 (5.2)		0.08
Infection sign or symptom, no. (%)					
Pneumonia	23 (8.6)	9 (6.7)	14 (10.4)		0.37
Sepsis	12 (4.5)	7 (5.2)	5 (3.7)		0.78
Polymicrobial infection	221 (82.2)	110 (81.5)	111 (82.8)		0.90
Laboratory finding‡					
Leukocyte count, 10^9^ cells/L (range)	8.2 (5.9–11.8)	8.2 (6.2–12.5)	7.8 (5.9–11.1)		0.52
Hemoglobin concentration, g/dL (range)	9.5 (8.4–10.5)	9.8 (8.8–11.0)	9.0 (8.1–10.2)		<0.01
Platelet count, 10^9^/L (range)	169.5 (82.0–268.0)	161.0 (84.0–263.5)	170.5 (80.0–271.0)		0.48
Erythrocyte sedimentation rate, mm/h (range)	49.0 (16.0–79.0)	52.0 (14.0–85.0)	39.5 (17.5–74.0)		0.28
Total bilirubin, mg/dL (range)	0.6 (0.3–1.4)	0.7 (0.4–1.6)	0.5 (0.3–1.2)		0.04
Serum creatinine, ng/mL (range)	0.6 (0.4–1.1)	0.6 (0.4–1.1)	0.6 (0.4–1.1)		0.78
C-reactive protein, mg/L (range)	51.4 (20.2–85.0)	63.6 (30.7–93.9)	35.2 (15.2–76.0)		<0.01
14-d mortality, no. (%)	32 (11.9)	20 (14.8)	12 (9.0)		0.20
In-hospital mortality, no. (%)	95 (35.3)	56 (41.5)	39 (29.1)		0.05
Location, no. (%)					<0.01
Ward A	27 (10.0)	3 (2.2)	24 (34.3)		
Ward B	39 (14.5)	3 (2.2)	36 (22.4)		
ICU 1	19 (7.1)	5 (3.7)	14 (26.1)		
ICU 2	3 (1.1)	3 (2.2)	0		
ICU 3	10 (3.7)	6 (4.4)	4 (3)		
Emergency department	8 (3)	8 (5.9)	0		
ICU 4	4 (1.5)	2 (1.5)	2 (1.5)		
ICU 5	36 (13.4)	21 (15.6)	15 (11.2)		
ICU 6	33 (12.3)	20 (14.8)	13 (9.7)		
ICU 7	9 (3.3)	5 (3.7)	4 (3)		
ICU 8	5 (1.9)	4 (3)	1 (0.7)		
NICU 1	15 (5.6)	7 (5.2)	8 (6)		
NICU 2	1 (0.4)	0	1 (0.7)		
Other	60 (22.3)	48 (35.6)	12 (9.0)		
Antimicrobial drug exposure, no. (%)					
Penicillin§	26 (9.7)	12 (8.9)	14 (10.4)		0.82
1st-generation cephalosporin	17 (6.3)	8 (5.9)	9 (6.7)		0.99
2nd-generation cephalosporin	19 (7.1)	10 (7.4)	9 (6.7)		0.99
3rd-generation cephalosporin	77 (28.6)	42 (31.1)	35 (26.1)		0.44
4th-generation cephalosporin	37 (13.8)	14 (10.4)	23 (17.2)		0.15
Aminoglycoside	25 (9.3)	12 (8.9)	13 (9.7)		0.98
Glycopeptide	106 (39.4)	52 (38.5)	54 (40.3)		0.86
Linezolid	11 (4.1)	8 (5.9)	3 (2.2)		0.22
Carbapenem	57 (21.2)	30 (22.2)	27 (20.1)		0.79
Tetracycline	12 (4.5)	6 (4.4)	6 (4.5)		0.99
Colistin	7 2.6)	7 (5.2)	0		0.02
Trimethoprim–sulfamethoxazole	32 (11.9)	20 (14.8)	12 (9.0)		0.20
Lincosamide	17 (6.3)	8 (5.9)	9 (6.7)		0.99
Macrolide	16 (5.9)	10 (7.4)	6 (4.5)		0.45
Fluoroquinolone	98 (36.4)	45 (33.3)	53 (39.6)		0.35

*ICU, intensive care unit; NICU, neonatal ICU. †May be multiple. ‡Reference ranges: leukocyte count, 4.0–10.8 × 10^9^ cells/L; hemoglobin concentration, 13.0–17.4 g/dL for adult male and 11.7–16.0 g/dL for adult female; platelet count, 150–400 × 10^9^/L; erythrocyte sedimentation rate, 0–15 mm/h for male and 0–20 mm/h for female; total bilirubin, 0.5–1.8 mg/dL for adult male, 0.4–1.5 mg/dL for adult female; serum creatinine, 0.68–1.19 ng/mL for adult male and 0.49–0.91 ng/mL for adult female; C-reactive protein, 0–8 mg/L. §Includes aminopenicillin, β-lactam/β-lactamase inhibitor.

### Surveillance Studies

We isolated 12 *Elizabethkingia* strains; all were derived from the 3 study wards. Seven *E. anophelis* isolates (4 from water taps in ICU 1, 2 from washbasins in ICU 1, and 1 from the suction regulator in ward A), 3 *E. miricola* isolates (3 from washbasins in ward A), and 2 *E. meningoseptica* isolates (1 from the mechanical ventilator after removal from a patient and 1 from the suction regulator in ward B) were identified by surveillance cultures.

**Figure 3:**
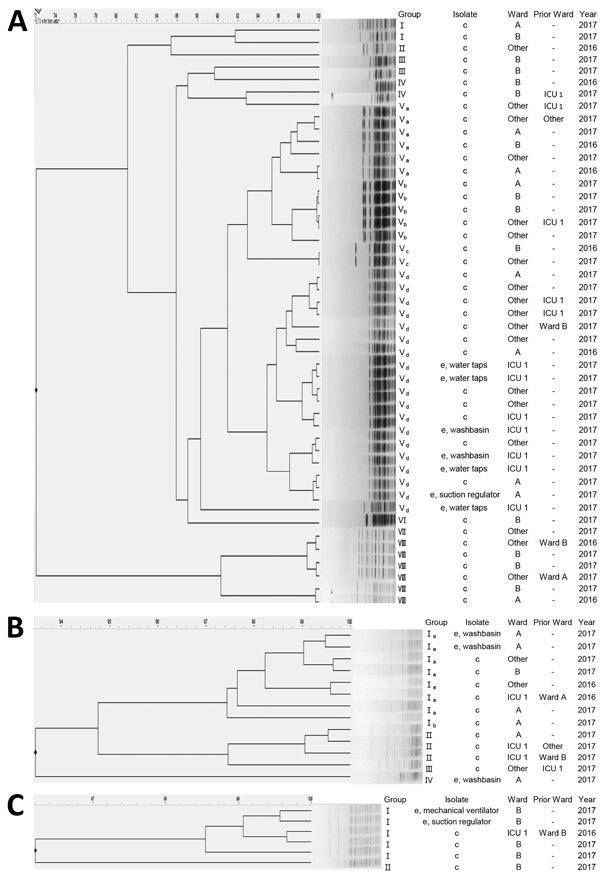
Pulsed-field gel electrophoresis dendrograms for 54 clinical isolates and 12 environmental isolates of *Elizabethkingia* species, Seoul, South Korea, 2017. *E. anophelis* (40 clinical isolates and 7 environmental isolates) showed 8 pulsotypes (A), *E. miricola* (10 clinical isolates and 3 environmental isolates) pulsotypes (B), and *E. meningoseptica* (4 clinical isolates and 2 environmental isolates) 2 pulsotypes (C). c, clinical; e, environmental; ICU, intensive care unit. Scale bar indicates percent relatedness.

We conducted PFGE typing on 54 clinical isolates (40 *E. anophelis*, 10 *E. miricola*, and 4 *E. meningoseptica*) from inpatients and 7 *E. anophelis*, 3 *E. miricola*, and 2 *E. meningoseptica* isolates from environmental samples. PFGE patterns showed that *E. anophelis* isolates belonged to 8 different PFGE groups, *E. miricola* to 4 groups, and *E. meningoseptica* to 2 groups (Figure 3). Five patients with *E. anophelis* (1 patient from ward B and 4 patients from other locations) had a history of admission to ICU 1 (Figure 3, panel A). Of patients from other locations, 1 had a history of admission to ward A and 2 had histories of admission to ward B. One patient in ICU 1 had moved from ward A, where the major cluster of environmental samples was isolated (Figure 3, panel B). Similarly, 1 patient in ICU 1 was transferred from ward B (Figure 3, panel C). This patient’s history of ward transfers suggests that transmission of the bacteria from patient to patient might be a cause of spreading. However, we cannot rule out the existence of other environmental sources.

We conducted PCR on 30 *Anopheles sinensis*, 8 *Culex tritaeniorhynchus*, and 3 *Aedes vexans* mosquitoes and on surveillance cultures collected from the midgut of mosquitoes. All yielded negative results.

### Epidemiologic Results Before and After PS Matching

Of the 6,583 patients potentially exposed on the 3 study wards, 74 were colonized or infected with *Elizabethkingia* species (Table 3). Case-patients and control-patients differed significantly in the proportion of hospitalized wards. The case-patient group, had higher admission rates to ward A and ward B, whereas control-patients had a higher rate of admission to ICU 1. Furthermore, case-patients had a significantly longer stay in the 3 study wards than did control-patients. In the 3 study wards, case-patients spent a median of 55 days (IQR 20–131 days), significantly longer than that of control patients (3 days [IQR 2–8 days]; p<0.001).

**Table 3:** Variables possibly associated with acquisition of *Elizabethkingia* species, before and after propensity score matching, in a tertiary teaching hospital, Seoul, South Korea

Variable	Before matching				After matching		
Case-patients, n = 74	Control-patients, n = 6,509	p value	Case-patients, n = 52	Control-patients, n = 156	p value
Ward, no. (%)			<0.01				0.54
Ward A	24 (32.4)	587 (9.0)			21 (40.4)	76 (48.7)	
Ward B	36 (48.6)	2,607 (40.1)			25 (48.1)	62 (39.7)	
Intensive care unit 1	14 (18.9)	3,315 (50.9)			6 (11.5)	18 (11.5)	
Period of admission, no. (%)			0.26				0.79
2016 Jan–Mar	14 (18.9)	1,073 (16.5)			6 (11.5)	32 (20.5)	
2016 Apr–Jun	14 (18.9)	1,172 (18.0)			10 (19.2)	27 (17.3)	
2016 Jul–Sep	11 (14.9)	1,078 (16.6)			11 (21.2)	30 (19.2)	
2016 Oct–Dec	16 (21.6)	1,141 (17.5)			6 (11.5)	13 (8.3)	
2017 Jan–Mar	15 (20.3)	1,058 (16.3)			10 (19.2)	27 (17.3)	
2017 Apr–Jun	4 (5.4)	987 (15.2)			9 (17.3)	27 (17.3)	
Median stay in 3 wards, d (range)	55 (20–131)	3 (2–8)	<0.01		32 (6–59)	20 (5–49)	0.27
Median age, y (range)	66.5 (59.0–76.0)	67.0 (57.0–75.0)	0.72		63.5 (57.5–73.0)	66.5 (54.0–77.0)	0.79
Sex, no. (%)			0.44				0.63
M	44 (59.5)	4,197 (64.5)			28 (53.8)	76 (48.7)	
F	30 (40.5)	2,312 (35.5)			24 (46.2)	80 (51.3)	
Median Charlson comorbidity index (range)	6 (4.0–7.0)	5 (4.0–7.0)	0.14		6 (4.0–6.5)	6 (3.0–7.0)	0.99
Comorbidities, no. (%)*							
Solid-organ tumor	18 (24.3)	1,766 (27.1)	0.68		11 (21.2)	53 (34.0)	0.12
Diabetes mellitus	11 (14.9)	894 (13.7)	0.91		7 (13.5)	21 (13.5)	0.99
Chronic pulmonary disease	9 (12.2)	207 (3.2)	<0.01		5 (9.6)	9 (5.8)	0.52
Chronic kidney disease	11 (14.9)	618 (9.5)	0.17		8 (15.4)	16 (10.3)	0.45
Hematologic malignancy	6 (8.1)	164 (2.5)	0.01		4 (7.7)	9 (5.8)	0.87
Dementia	3 (4.1)	147 (2.3)	0.52		2 (3.8)	8 (5.1)	0.99
Connective tissue disease	3 (4.1)	253 (3.9)	0.99		3 (5.8)	9 (5.8)	0.99
Mild liver disease	2 (2.7)	82 (1.3)	0.56		1 (1.9)	3 (1.9)	0.99
Steroid use	23 (31.1)	562 (8.6)	<0.01		14 (26.9)	34 (21.8)	0.57
Mechanical ventilation	72 (97.3)	1,258 (19.3)	<0.01		50 (96.2)	62 (39.7)	<0.01
Antimicrobial exposure, no. (%)							
Penicillin†	5 (6.8)	393 (6.0)	0.99		2 (3.8)	15 (9.6)	0.31
1st-generation cephalosporin	2 (2.7)	445 (6.8)	0.24		1 (1.9)	11 (7.1)	0.30
2nd-generation cephalosporin	2 (2.7)	184 (2.8)	0.99		2 (3.8)	2 (1.3)	0.56
3rd-generation cephalosporin	26 (35.1)	1,089 (16.7)	<0.01		15 (28.8)	40 (25.6)	0.79
4th-generation cephalosporin	0	1	0.99		0	0	NA
Aminoglycoside	13 (17.6)	118 (1.8)	<0.01		10 (19.2)	12 (7.7)	0.04
Glycopeptide	40 (54.1)	481 (7.4)	<0.01		27 (51.9)	40 (25.6)	<0.01
Linezolid	6 (8.1)	40 (0.6)	<0.01		5 (9.6)	5 (3.2)	0.13
Carbapenem	42 (56.8)	416 (6.4)	<0.01		27 (51.9)	42 (26.9)	<0.01
Tetracycline	12 (16.2)	58 (0.9)	<0.01		5 (9.6)	7 (4.5)	0.30
Trimethoprim–sulfamethoxazole	17 (23.0)	245 (3.8)	<0.01		11 (21.2)	15 (9.6)	0.05
Lincosamide	7 (9.5)	40 (0.6)	<0.01		2 (3.8)	2 (1.3)	0.56
Macrolide	1 (1.4)	286 (4.4)	0.32		1 (1.9)	4 (2.6)	0.99
Fluoroquinolone	43 (58.1)	871 (13.4)	<0.01		30 (57.7)	45 (28.8)	<0.01
Other	5 (6.8)	100 (1.5)	<0.01		4 (7.7)	5 (3.2)	0.33

*May be multiple. †Includes aminopenicillin, β-lactam/β-lactamase inhibitor.

We conducted PS matching to adjust baseline demographics and clinical variables between the case-patient and control-patient groups. PS matching resulted in 52 matched pairs at a 1:3 ratio. After matching, we included 52 of 74 case-patients in the analysis. Confounding variables were well balanced in the 2 groups, including hospitalization ward and the period of admission and length of stay in the 3 study wards. Furthermore, the 2 comorbidities after PS matching did not differ significantly because there was an adjustment to the proportions of admission to ward A, which had high rates of patients with hematologic malignancy, and ward B, which had high rates of patients with chronic pulmonary disease. However, use of mechanical ventilation still differed significantly after PS matching (p<0.001).

We used univariate and multivariate analyses with conditional logistic regression to identify independent risk factors (Table 4). Only use of mechanical ventilation (adjusted odds ratio [OR] 50.44 [95% CI 6.74–377.48]; p<0.001) was associated with the acquisition of *Elizabethkingia* species.

**Table 4:** Results of univariate and multivariate analysis using conditional logistic regression of risk factors for the acquisition of *Elizabethkingia* species at a tertiary teaching hospital after propensity score matching, Seoul, South Korea*

Variable	Univariate analysis			Multivariate analysis†	
OR (95% CI)	p value	OR (95% CI)	p value
Ward					
Ward A	Reference				
Ward B	0.87 (0.41–1.79)	0.70			
Intensive care unit 1	0.69 (0.18–2.22)	0.56			
Period of admission					
2016 Jan–Mar	Reference				
2016 Apr–Jun	8.03 (0.79–81.98)	0.08			
2016 Jul–Sep	8.87 (0.74–105.84)	0.08			
2016 Oct–Dec	10.62 (0.64–176.77)	0.10			
2017 Jan–Mar	8.87 (0.97–81.16)	0.05			
2017 Apr–Jun	8.34 (0.71–98.76)	0.09			
Median stay in 3 wards, d	1.01 (0.99–1.02)	0.25			
Age, y	1.00 (0.98–1.02)	0.87			
Male sex	1.06 (0.55–2.02)	0.87			
Charlson comorbidity index	0.97 (0.85–1.12)	0.69			
Comorbidities‡					
Solid organ tumor	0.48 (0.21–1.08)	0.08			
Diabetes mellitus	0.89 (0.34–2.31)	0.81			
Chronic pulmonary disease	1.85 (0.55–6.28)	0.32			
Chronic kidney disease	1.52 (0.60–3.85)	0.38			
Hematologic malignancy	1.00 (0.25–4.00)	0.99			
Dementia	0.57 (0.12–2.77)	0.49			
Connective tissue disease	0.80 (0.21–3.05)	0.75			
Mild liver disease	1.00 (0.10–9.61)	0.99			
Steroid use	1.55 (0.62–3.89)	0.35			
Mechanical ventilation	64.54 (8.76–475.30)	<0.01		50.44 (6.74–377.48)	<0.01
Antimicrobial exposure					
Penicillin§	0.32 (0.07–1.54)	0.16			
1st-generation cephalosporin	0.29 (0.03–2.88)	0.29			
2nd-generation cephalosporin	3.00 (0.42–21.30)	0.27			
3rd-generation cephalosporin	0.97 (0.46–2.01)	0.93			
4th-generation cephalosporin	NA				
Aminoglycoside	3.18 (1.21–8.31)	0.02		2.30 (0.62–8.47)	0.21
Glycopeptide	3.96 (1.82–8.63)	<0.01		1.72 (0.50–5.86)	0.39
Linezolid	8.84 (0.97–80.69)	0.05			
Carbapenem	4.16 (1.99–8.72)	<0.01		1.63 (0.55–4.85)	0.38
Tetracycline	1.65 (0.42–6.43)	0.47			
Trimethoprim/sulfamethoxazole	2.11 (0.90–4.91)	0.09			
Lincosamide	6.00 (0.54–66.17)	0.14			
Macrolide	0.75 (0.08–6.71)	0.80			
Fluoroquinolone	3.42 (1.70–6.87)	<0.01		2.01 (0.71–5.69)	0.19
Other	2.09 (0.48–9.03)	0.33			

*OR, odds ratio; NA, not available. †Only variables with p<0.05 in the univariate model were included in the multivariate model. ‡May be multiple. §Includes aminopenicillin, β-lactam/β-lactamase inhibitor.

Only 30 patients were classified as true pathogen cases (Table 5). In the hospital mortality group, the median total hospitalization stay was longer than that of the nonhospital mortality group (77.5 vs. 38.5 days; p = 0.04). More case-patients were treated with carbapenem or trimethoprim/sulfamethoxazole than were those in the nonhospital mortality group (5 vs. 2 case-patients; p = 0.05).

**Table 5:** Results of univariable and multivariable analyses of risk factors for in-hospital mortality of patients with a true pathogen of *Elizabethkingia* in a tertiary teaching hospital, Seoul, South Korea

In-hospital mortality	Total, n = 30	Survived, n = 20	Died, n = 10	p value
Median age, y (range)	68.5 (61.0–80.0)	69.5 (60.5–79.5)	66.5 (63.0–80.0)	0.86
Male sex, no. (%)	19 (63.3)	11 (55.0)	8 (80.0)	0.35
Patients from the 3 study wards, no. (%)	7 (23.3)	5 (25.0)	2 (20.0)	0.99
Nosocomial infection, no. (%)	29 (96.7)	19 (95.0)	10 (100.0)	0.99
Median Charlson comorbidity index (range)	6 (5.0–9.0)	6 (4.5–7.5)	6 (5.0–9.0)	0.93
Clinical condition				
Median hospitalization day of acquisition (range)	26.5 (13.0–58.0)	20.5 (12.0–32.0)	52.5 (26.0–81.0)	0.03
Median length of hospitalization, d (range)	47.5 (29.0–89.0)	38.5 (27.5–67.5)	77.5 (54.0–210.0)	0.04
Mechanical ventilation, no. (%)	24 (80.0)	15 (75.0)	9 (90.0)	0.63
Steroid use, no. (%)	14 (46.7)	9 (45.0)	5 (50.0)	0.99
Antimicrobial treatment, no. (%)				
Penicillin*	5 (16.7)	3 (15.0)	2 (20.0)	0.99
1st-generation cephalosporin	3 (10)	2 (10.0)	1 (10.0)	0.99
2nd-generation cephalosporin	5 (16.7)	4 (20.0)	1 (10.0)	0.86
3rd-generation cephalosporin	8 (26.7)	4 (20.0)	4 (40.0)	0.47
4th-generation cephalosporin	7 (23.3)	4 (20.0)	3 (30.0)	0.88
Aminoglycoside	1 (3.3)	1 (5.0)	0	0.99
Glycopeptide	13 (43.3)	6 (30.0)	7 (70.0)	0.09
Linezolid	4 (13.3)	2 (10.0)	2 (20.0)	0.85
Carbapenem	7 (23.3)	2 (10.0)	5 (50.0)	0.05
Tetracycline	8 (26.7)	7 (35.0)	1 (10.0)	0.30
Colistin	3 (10)	0	3 (30.0)	0.05
Trimethoprim/sulfamethoxazole	7 (23.3)	2 (10.0)	5 (50.0)	0.05
Lincosamide	5 (16.7)	1 (5.0)	4 (40.0)	0.06
Macrolide	1 (3.3)	0	1 (10.0)	0.72
Fluoroquinolone	9 (30)	4 (20.0)	5 (50.0)	0.21
Other	5 (16.7)	2 (10.0)	3 (30.0)	0.39

*Includes aminopenicillin, β-lactam/β-lactamase inhibitor.

## Discussion

The incidence of infection with *Elizabethkingia* species has increased in recent years in many countries (*14**,**16**–**19*). Furthermore, a large-scale outbreak was reported in community settings in the United States (*28*).

In previous studies, the reported annual incidence of *E. meningoseptica* ranged from 0.007 to 0.399 cases per 1,000 admissions (*19**,**29*). We reported the antimicrobial resistance mechanisms and susceptibility rates of *Elizabethkingia* species isolated from Severance Hospital in 2010 (*11*) and 2016 (*7*). Recently, the incidence of isolation in this hospital increased significantly, from 0.02 to 0.88 per 1,000 admissions during 2009–2017, mainly in the 3 study wards.

We analyzed the risk factors associated with the acquisition of *Elizabethkingia* species after controlling for other confounding variables. Using multivariate analysis, we found that the probability of acquiring *Elizabethkingia* species was significantly influenced by whether a patient received mechanical ventilation, even after PS matching and adjustment for other variables. Although some previous studies have suggested that mechanical ventilators could be a risk factor for colonization or infection with *Elizabethkingia* species, they did not provide a statistical analysis and included only a small number of patients (*16**,**30*). In our study, a total of 214 (79.6%) patients from among the 269 patients seen during January 2009–June 2017 received mechanical ventilation, as did 72 (97.3%) of 74 case-patients admitted to the 3 study wards during January 2016–June 2017. We included a large number of cases and tried to control for confounding variables using PS matching, thus ensuring that mechanical ventilation is related to the acquisition of *Elizabethkingia* species.

Water or water-related equipment can serve as a waterborne pathogen reservoir in the hospital environment (*31*). Previous studies also have associated a water source with acquisition of *Elizabethkingia* because of the bacterium’s ability to form a biofilm in moist environments. Balm et al. reported the infections of 5 patients in 1 outbreak with *E. meningoseptica* were related to a hand hygiene sink aerator (*14*). Moore et al. identified 30 patients as having acquired *E. meningoseptica* during an outbreak; at least 10 of these infections were associated with 5 environmental samples isolated from sinks (*13*). *Elizabethkingia* can spread from a humid environment to the surface of medical devices or dry materials by the hands of hospital staff or patients (*32*). In our current study, all 12 environmental isolates shared identical PFGE patterns with clinical isolates. Our finding is consistent with prior reports that *Elizabethkingia* acquisition might be related to water sources within the hospital environment. In contrast, we could not find any evidence that the local mosquitoes of South Korea act as vehicles of *Elizabethkingia* transmission.

As extended-spectrum β-lactamase–producing bacteria have increased, the use of carbapenems has inevitably increased. Previous reports have suggested that antimicrobial selective pressure may increase the prevalence of bacteria with natural resistance to carbapenems, such as *Elizabethkingia* species (*33**,**34*). Unlike in our univariate analysis, our multivariate analysis showed no association between antimicrobial exposure to carbapenem and the acquisition of *Elizabethkingia*. One possible explanation for this finding could be that host factors are a more important selection factor than the antimicrobial drug in the selection of this strain. Another explanation could be that our data lacked the statistical power to detect differences in *Elizabethkingia* acquisition by antimicrobial exposure to carbapenem.

Difficulties in eradicating and terminating outbreaks of *Elizabethkingia* caused by a strong biofilm biotype have been reported (*13**,**14**,**35*). Furthermore, the failure of 1,000-ppm sodium hypochlorite and posthandwashing alcohol gel has been documented (*34*). The acquisition risk can be reduced by regular sink flushing and improvements to the workflow that minimize contamination (*13*). Fortunately, we succeeded in eliminating *Elizabethkingia* species in ward B in September 2017. After all structures from which bacteria were isolated were dismantled, the outer surface and inner spaces were cleaned. A sheer force was applied using a sodium hypochlorite solution and a brush, then structures were reinstalled. In ward B, no new patient acquisitions of *Elizabethkingia* occurred after this effort. These findings also support the possibility that *Elizabethkingia* acquisition may be related to water source and the contaminated devices. However, a previous study documented that *Elizabethkingia* species have been re-isolated after a month, even after all contaminated devices were replaced (*14*). Continuous monitoring, including surveillance culture systems and education for medical staff, may be more important than decontamination in reducing the acquisition of and infection with *Elizabethkingia*.

Among our data on treatment outcomes for true pathogen cases, none of the antimicrobial agents used after reporting the culture results were related to reducing in-hospital death. Alternatively, patients treated with carbapenem or trimethoprim/sulfamethoxazole had significantly higher in-hospital death rates. However, this analysis included only 30 true pathogen cases, and results could not be adjusted for other potential confounding factors.

The retrospective and single-center nature of the study limited our results. Thus, selection bias might exist in the tests performed on environmental samples and mosquito samples, and we could not identify the species of bacteria that were not stored. It is also difficult to perform additional surveillance cultures in the hospital setting because we have conducted elimination procedures to manage bacterial spread. However, we tried to analyze risk factors for *Elizabethkingia* acquisition by minimizing the selection bias using a PS-matched study and multivariate analysis.

Even after controlling for potential biases using PS matching analysis, we found mechanical ventilation to be an independent risk factor for the acquisition of *Elizabethkingia* species. Because *Elizabethkingia* infection has a high rate of death and is difficult to eliminate, intensive prevention of contamination is needed.
