# Correction: Vol. 25, No. 1

**DOI:** 10.3201/eid2502.C42502

**Published:** 2019-02

**Keywords:** errata, erratum, correction

Two locations in Figure 1 were shown incorrectly in Risk Factors for *Elizabethkingia* Acquisition and Clinical Characteristics of Patients, South Korea (M.H. Choi et al.). The corrected figure is shown, and the article has been corrected online (https://wwwnc.cdc.gov/eid/article/25/1/17-1985_article).

